# THE EFFECTS OF A DECADE OF CHANGE IN THE YOUNG-OLD POPULATION IN A MARGINAL COMMUNITY IN JAPAN

**DOI:** 10.1093/geroni/igz038.3191

**Published:** 2019-11-08

**Authors:** Yuichi Watanabe

**Affiliations:** Musashino University, Nishitokyo, Tokyo, Japan

## Abstract

Population aging is occurring throughout the world, with an estimated 82 countries expected to have more than 20% of their population age 65 or older by 2050. Communities in which these older adults live face significant challenges in maintaining residential services. Japan is on the leading edge of this phenomenon. In Japan, marginal communities are defined as those where over 50% of the population is 65 years of age or older and face difficulties maintaining the traditional mutual support system in the community. The old-old population might have vulnerabilities because of a lack of resources for daily living. Documenting the changes in these communities over time may help to identify solutions to how to be an inclusive community. we examine the effects of a decade of change using surveys completed in a marginal community in Japan in 2009 and 2019. Data were collected from a cohort of 65 to 74 year olds in 2009 (n=45) and 75 to 84 year olds in 2019 (n=26) in one marginal community. Analysis by Fisher’s exact test shows decreasing the exchange of information and decreasing discussion of the future of the community with family and relatives over time. In 2009, respondents surveyed brought up issues such as the shortage of care services and lack of bank facilities, while in 2019 these issues were not mentioned. Results suggest that decisions made regarding the viability of those communities should include input from the elderly to optimize the effects of aging in place.

